# Using network pharmacology and molecular docking verification to explore the mechanism of ursolic acid in the treatment of osteoporosis

**DOI:** 10.1097/MD.0000000000032222

**Published:** 2022-12-09

**Authors:** Bowen Yang, Qiuwen Zhu, Xiaodong Wang, Jingxin Mao, Shuqing Zhou

**Affiliations:** a Department of Orthopedics, Jiangjin Central Hospital of Chongqing, Chongqing, China; b Department of Nephrology, Jiangjin Central Hospital of Chongqing, Chongqing, China; c Department of Pharmacology, Chongqing Medical and Pharmaceutical College, Chongqing, China; d College of Pharmaceutical Sciences, Southwest University, Chongqing, China.

**Keywords:** molecular docking, molecular mechanism, network pharmacology, osteoporosis, ursolic acid

## Abstract

Whether ursolic acid is an effective drug in treatment of osteoporosis (OP) and how it exhibit activity effect on OP is unclear. To investigated the potential molecular mechanism of ursolic acid in the treatment of OP and figured out its possible mechanism is necessary. The target genes of ursolic acid were screened by using the database of traditional chinese medicine systems pharmacology, PubMed database and UniProt database. OP-related target genes were searched by GeneCards database, and utilized online mapping tool to obtain common target genes of component-disease. String database was used to construct a protein-protein interaction (PPI) network of component-disease common target genes and perform topological analysis to screen core target genes. DAVID database was performed gene ontology (GO) functional annotation and Kyoto Encyclopedia of Genes and Genomes (KEGG) pathway enrichment analysis for component-disease shared target genes. Using the core target protein as the receptor and ursolic acid as the ligand, the molecular docking was performed using AutoDockVina 1.1.2 software. A total of 52 ursolic acid-related target genes and 4657 OP-related target genes were excavated, with a total of collective 43 target genes. The above-mentioned PPI network with shared target genes contains 43 nodes and 510 edges, with an average node degree value of 23.32. A total of 24 core target genes were obtained, mainly including tumor protein p53 (TP53), vascular endothelial growth factor A (VEGFA), interleukin-6 (IL6), tumor necrosis factor (TNF), caspase3 (CASP3), matrix metallo protein (MMP9), transcription factor AP-1 (JUN), activator of transcription 3 (STAT3), mitogen-activated protein kinase 8 (MAPK8), and prostaglandin endoperoxidase 2 (PTGS2), respectively. According to KEGG enrichment analysis, there are 126 treatment of OP signaling pathway were enriched. GO enrichment analysis revealed that 313 biological processes were identified. The molecular docking result showed that the binding energies were all lower than −5 kcal/mol, indicating strong binding activity to the protein by the 6 core target gene. The therapeutic effect of ursolic acid on OP may be achieved by regulating TP53, JUN, IL6, VEGFA, CASP3, and MAPK8 genes, respectively. It exhibits possible biological function in the treatment of OP mainly involve positive regulation of apoptotic process, response to drug, incytoplasm, cytosol, protein binding, identical protein binding. Its mechanism may related to multiple therapeutic targets and signaling pathways such as cancer pathway, hepatitis B, and TNF signaling pathway.

## 1. Introduction

Osteoporosis (OP) is an inflammatory metabolic disease with a complex occurrence and development process, which is closely related to multiple genes and signaling pathways in the body. With the increase of population growth and aging, the incidence of OP is also increasing year by year, which has become a major public health problem in the world.^[1]^ There are currently 200 million women with OP in the world, and about 30% of the elderly over 50 years old have 1 or more fractures. The situation is very serious. In addition, the disease may also induce other diseases, causing a heavy economic burden to the patient’s family and society.^[2]^ The elderly are the main patient population of OP. At present, most of the clinical drugs for the treatment of OP are chemical drugs that regulate bone metabolism, but they may cause a series of adverse reactions such as kidney damage, which limits their clinical application.^[3]^ Since there are no obvious symptoms in the early stage of the disease, once it is diagnosed, it is relatively late, and even life-threatening, finally resulting in serious and irreversible consequences. OP belongs to the category of “bone atrophy” in the Traditional Chinese Medicine (TCM) which is to invigorate the kidney and replenish the essence, activate blood and remove blood stasis as the basic principle.^[4]^ However, the relevant molecular mechanism of TCM treatment of OP has not been fully elucidated, which restricts the further transformation of related TCM theories to clinical practice. Most of the current new drug screening ideas focus on the targeted therapy and mechanism of drugs on a certain key protein and molecule. Ursolic acid has anti-oxidative,^[5]^ anti-inflammatory,^[6]^ anti-viral,^[7]^ anti-tumor^[8,9]^ and other effects, and is widely distributed in nature. Relevant studies have shown that ursolic acid may significantly improve kidney damage.^[10]^ The TCM theory believes that kidney deficiency is the main pathogenesis of OP, advocates that the treatment of OP should be paralleled with the protection of renal function.^[11]^ Therefore, according to the theory of “invigorating the kidney and strengthening bones” in TCM, ursolic acid may have a potential OP improvement effect. Network pharmacology is to use the achievements of computer science, molecular biology, pharmacy and other disciplines to carry out multi-gene, multi-target and multi-channel systematic research on the potential targets and pharmacological effects of TCM ingredients, which can provide information and new ideas for the modernization of TCM.^[12]^ Molecular docking is a theoretical simulation method that predicts the binding mode and affinity of the receptor through the interaction between the receptor and the drug molecule.^[13]^ Combining the network pharmacology method and molecular docking technology to preliminarily explore the potential targets and pathways of ursolic acid in the treatment of OP in the study. It may provided a basis for further elucidation of the molecular mechanism of ursolic acid in the treatment of OP.

## 2. Methods

### 2.1. Extraction of target genes of ursolic acid

The potential target proteins of ursolic acid were retrieved through the database of traditional chinese medicine systems pharmacology (https://old.tcmsp-e.com/tcmsp.php), and the potential target proteins of ursolic acid were retrieved from the PubMed database (https://pubmed.ncbi.nlm.nih.gov) for structural alignment. Through the UniProt database (https://www.uniprot.org), the proteins corresponding to the above targets were converted into human genes, and a database of ursolic acid monomer compounds and their target genes was constructed.

### 2.2. Screening of OP-related target genes

Using “osteoporosis” as the keyword, a human gene search was performed in the GeneCards database (https://www.genecards.org) to obtain the disease target genes of OP.

### 2.3. Screening of component-disease common target genes

The two groups of targets screened in 2.1 and 2.2 were entered into the online mapping tool (http://bioinformatics.psb.ugent.be/cgi-bin/liste/Venn/calculate_venn.htpl) to draw venn diagram for component-disease common target genes.

### 2.4. Construction and topology analysis of protein-protein interaction network of shared target genes

Import the common target genes of component-disease into the String database (https://string-db.org/cgi/input.pl), set the biological species as “homosapiens (Homo sapiens),” and construct proteins with common target genes -protein interaction relationship (PPI) network, and import the construction results into Cytoscape 3.8.0 software for visual display. In the visual network diagram, the common target gene is represented by a node. The darker the color and the larger the shape, the greater the degree of the corresponding target gene, that is, the more important the target gene is; the edge represents the common target gene. The thicker the line, the greater the combination score between target genes, that is, the more significant the correlation between target genes and target genes.^[14]^ Topological analysis of the PPI network was performed with the help of the “networkanalyzer” tool in the Cystoscape 3.8.0 software. The median value of 3 parameters, including degree value, betweenness centrality, and closeness centrality, is used as the reference index. Among them, betweenness centrality represents the number of shortest paths passing through a node, the greater the betweenness centrality, the greater the influence of the node^[15]^; the closeness centrality represents the average distance between the node and other nodes in the network, and the closeness to the center, the higher the degree of nodes, the better the information flow^[16]^; the target gene with a degree value greater than the average degree value is selected as the core target gene, and the degree value, betweenness centrality and closeness centrality are all ranked. The topology analysis results of the top 10 core target genes are displayed.

### 2.5. Gene ontology functional annotation and KEGG pathway enrichment analysis

GO functional annotation and KEGG pathway enrichment analysis were performed on component-disease shared target genes using the DAVID database (https://david.ncifcrf.gov). Among them, GO functional annotation analysis mainly involves biological process (BP), molecular function (MF), cellular component (CC) functions. Set the species to “homosapiens” and the identifier to “official genesymbol (official name),” retain entries with a corrected *P* value less than .05, and sort them according to the corrected *P* value from small to large. Select the top 20 entries and import them into RStudio 3.6.3 software to visualize the enrichment results.

### 2.6. Component core target gene coding protein molecular docking test

Download the 3-dimensional structure of the protein encoded by the core target gene from the protein data bank database (https://www.rcsb.org), and save it in the “protein data bank” format as the protein receptor. Using AutoDockTools 1.5.6 to removes water molecules, separates proteins, adds non-polar hydrogen and other treatments to the protein receptor, calculates the gasteiger charge of its structure to ensure that the atoms follow the atomic type in autodock, and then saves it as a “pdbqt” format file. Download the 2-dimensional structure of ursolic acid from PubChem database (https://pubchem.ncbi.nlm.nih.gov), save it in “mol2” format, and utilized AutoDockTools 1.5.6 after hydrogenation, charging, detecting the central node of ligand molecule and searching and defining the rotatable bond, the software will save it as a “pdbqt” format file as the ligand. The receptor is set to be rigid and the ligand is set to be flexible, then Autodock vina 1.1.2 software was used for molecular docking of protein receptor encoded by core target gene and ursolic acid ligand. The binding ability of ligand and receptor is evaluated by binding energy. If the binding energy is less than 0, it means that ligand and receptor can spontaneously bind, and the smaller the value, the higher the binding activity.^[17]^ Pymol 2.4.0 software was used for visual display of docking results finally.

## 3. Results

### 3.1. Screening results of target genes related to ursolic acid and OP

**Figure 1.**
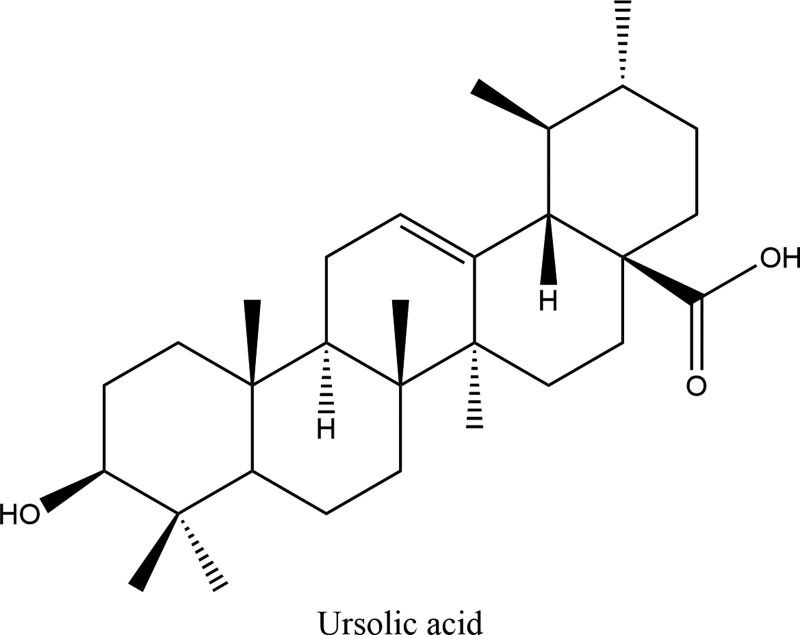
The 2D structure of ursolic acid. 2D = two-dimensional.

**Figure 2.**
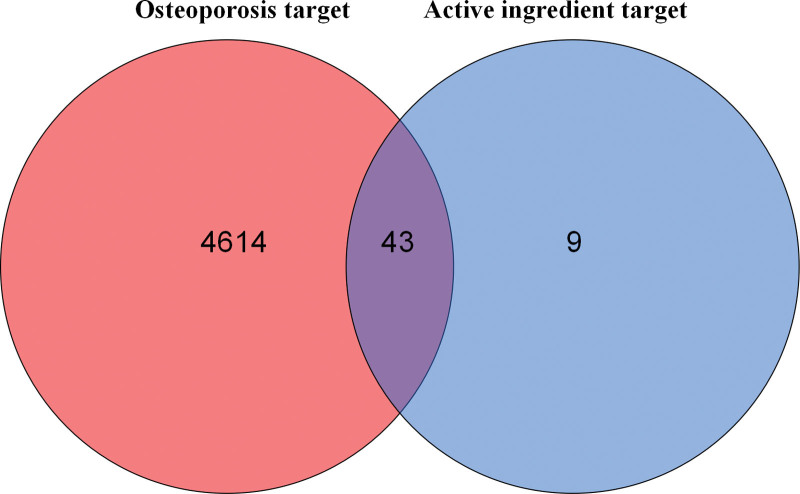
Venn diagram of the common target gene screening of ursolic acid and OP. OP = osteoporosis.

The molecular formula of ursolic acid is C_30_H_48_O_3_ (PubChem CID:64945) and its molecular weight is 498.737. Ursolic acid is a kind of triterpenoids in natural plants, the 2-dimensional structure of ursolic acid was presented in Figure 1. After searching, summarizing and deleting the duplicate, a total of 52 ursolic acid related target genes were obtained; 4657 OP related target genes were retrieved in genecards database, and 43 component disease target genes were obtained (Fig. 2).

### 3.2. Construction of PPI network of common target genes and screening results of core target genes

**Figure 3.**
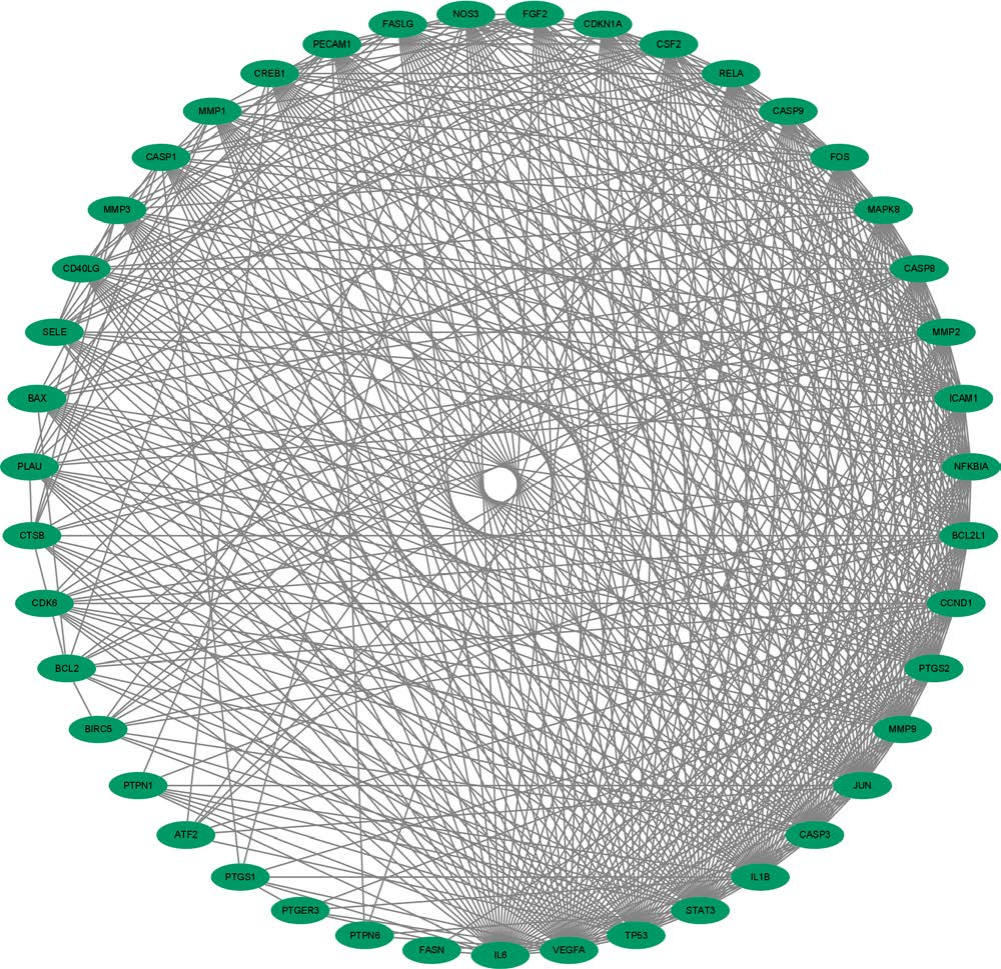
Component-PPI visualization network diagram of disease common target genes. PPI = protein-protein interaction.

The PPI visualization network of component disease common target genes was shown in Figure 3. The network has 43 nodes and 510 edges, and the average node degree is 23.32. There are no isolated nodes in the network, and the connectivity between nodes is good, indicating that ursolic acid may play its role in the treatment of OP through multi-target collaborative regulation. The results of network topology analysis show that there are 24 core target genes which node degree value is greater than the average degree value (23.32). The top 10 core target genes with degree value, intermediate centrality and near centrality are TP53, VEGFA, IL6, TNF, caspase3 (CASP3), MMP9, JUN, STAT3, MAPK8, and PTGS2 respectively. The results of topology analysis are shown in Table 1.

**Table 1:** Topological analysis results of core target genes of ursolic acid in the treatment of OP.

Core target gene	Degree value	Betweenness centrality	Closeness centrality
VEGFA	38	79.96	40.5
TP53	38	97.06	40.5
IL6	36	85.29	39.5
CASP3	36	65.39	39.5
JUN	36	66.26	39.5
MMP9	35	50.32	39.0
STAT3	35	47.80	39.0
TNF	35	38.35	39.0
MAPK8	35	40.74	39.0
PTGS2	33	38.68	38.0

CASP3 = caspase3, IL6 = interleukin-6, JUN = transcription factor AP-1, MAPK8 = mitogen-activated protein kinase 8, MMP9 = matrix metallo protein, PTGS2 = prostaglandin endoperoxidase 2, STAT3 = activator of transcription 3, TP53 = tumor protein p53, TNF = tumor necrosis factor, VEGFA = vascular endothelial growth factor A.

### 3.3. GO functional enrichment analysis results

**Figure 4.**
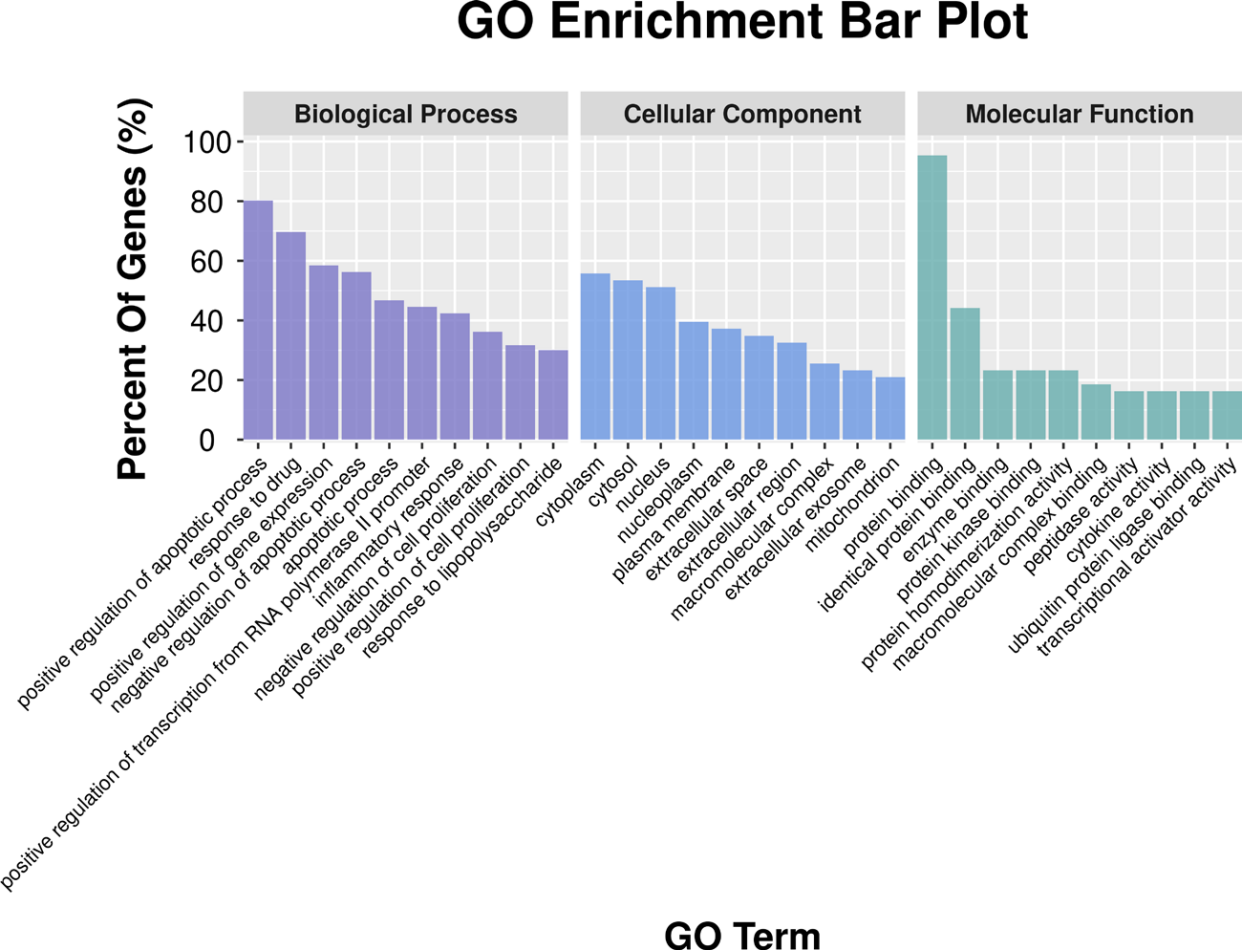
Bar plot of GO functional enrichment analysis. GO = gene ontology.

Screening was conducted according to the criterion that the corrected *P* value was less than .05, and a total of 313 GO functional items were enriched in BP (284), MF (58), and CC (28) items, respectively. Among them, BP is mainly involved in positive regulation of apoptotic process, response to drug, positive regulation of gene expression, negative regulation of apoptotic process, apoptotic process, positive regulation of transcription from RNA polymerase II promoter, inflammatory response, negative regulation of cell proliferation, positive regulation of cell proliferation, response to lipopolysaccharide etc. CC is mainly involved incytoplasm, cytosol, nucleus, nucleoplasm, plasma membrane, extracellular space, extracellular region, macromolecular complex, extracellular exosome, mitochondrion etc. MF is mainly involved in protein binding, identical protein binding, enzyme binding, protein kinase binding, protein homodimerization activity, macromolecular complex binding, peptidase activity, cytokine activity, ubiquitin protein ligase binding, transcriptional activator activity etc. The bubble plot of the GO functional enrichment analysis (top 20 after correction *P* value) is shown in Figure 4.

### 3.4. KEGG pathway enrichment analysis results

**Figure 5.**
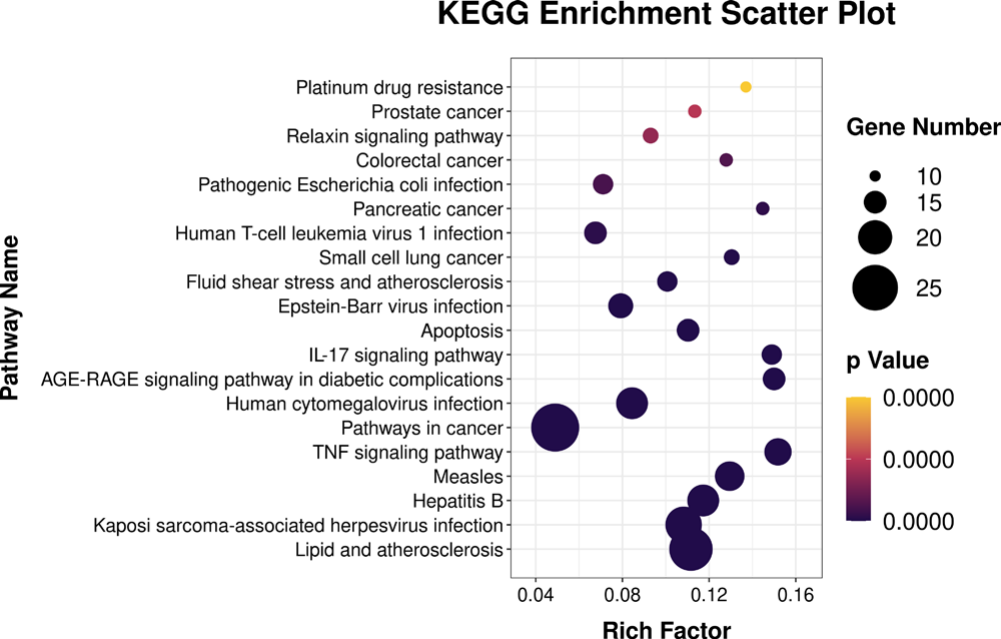
Bubble diagram of KEGG pathway enrichment analysis. KEGG = kyoto encyclopedia of genes and genomes.

Screening was performed according to the standard that the corrected *P* value was less than .05, and a total of 126 KEGG signaling pathways were enriched. The visual display results of the top 20 items with the corrected *P* value are shown in Figure 5, and the specific information is shown in Table 2. It was showed that the potential targets of ursolic acid in the treatment of OP mainly include hepatitis B, TNF signaling pathway, pathways in cancer, human cytomegalovirus infection (HTLV), AGE-RAGE signaling pathway in diabetic complications, IL-17 signaling pathway, apoptosis, human T-cell leukemia virus 1 infection, pancreatic cancer, pathogenic *Escherichia coli* infection, colorectal cancer, relaxin signaling pathway, prostate cancer, platinum drug resistance and many other pathways.

**Table 2:** KEGG enrichment analysis results of ursolic acid for OP treatment.

KEGG ID	Pathway name	Gene number	*P* value
hsa05417	Lipid and atherosclerosis	24	4.32E-26
hsa05167	Kaposi sarcoma-associated herpesvirus infection	21	4.12E-22
hsa05161	Hepatitis B	19	2.10E-20
hsa05162	Measles	18	5.68E-20
hsa04668	TNF signaling pathway	17	6.62E-20
hsa05200	Pathways in cancer	26	1.14E-19
hsa05163	Human cytomegalovirus infection	19	8.52E-18
hsa04933	AGE-RAGE signaling pathway in diabetic complications	15	2.72E-17
hsa04657	IL-17 signaling pathway	14	5.23E-16
hsa04210	Apoptosis	15	2.41E-15
hsa05169	Epstein-Barr virus infection	16	2.71E-14
hsa05418	Fluid shear stress and atherosclerosis	14	9.92E-14
hsa05222	Small cell lung cancer	12	6.67E-13
hsa05166	Human T-cell leukemia virus 1 infection	15	2.24E-12
hsa05212	Pancreatic cancer	11	3.16E-12
hsa05130	Pathogenic Escherichia coli infection	14	9.04E-12
hsa05210	Colorectal cancer	11	1.13E-11
hsa04926	Relaxin signaling pathway	12	2.90E-11
hsa05215	Prostate cancer	11	3.86E-11
hsa01524	Platinum drug resistance	10	7.93E-11

KEGG = kyoto encyclopedia of genes and genomes, OP = osteoporosis, TNF = tumor necrosis factor.

### 3.5. Molecular docking results of component-core target protein

**Figure 6.**
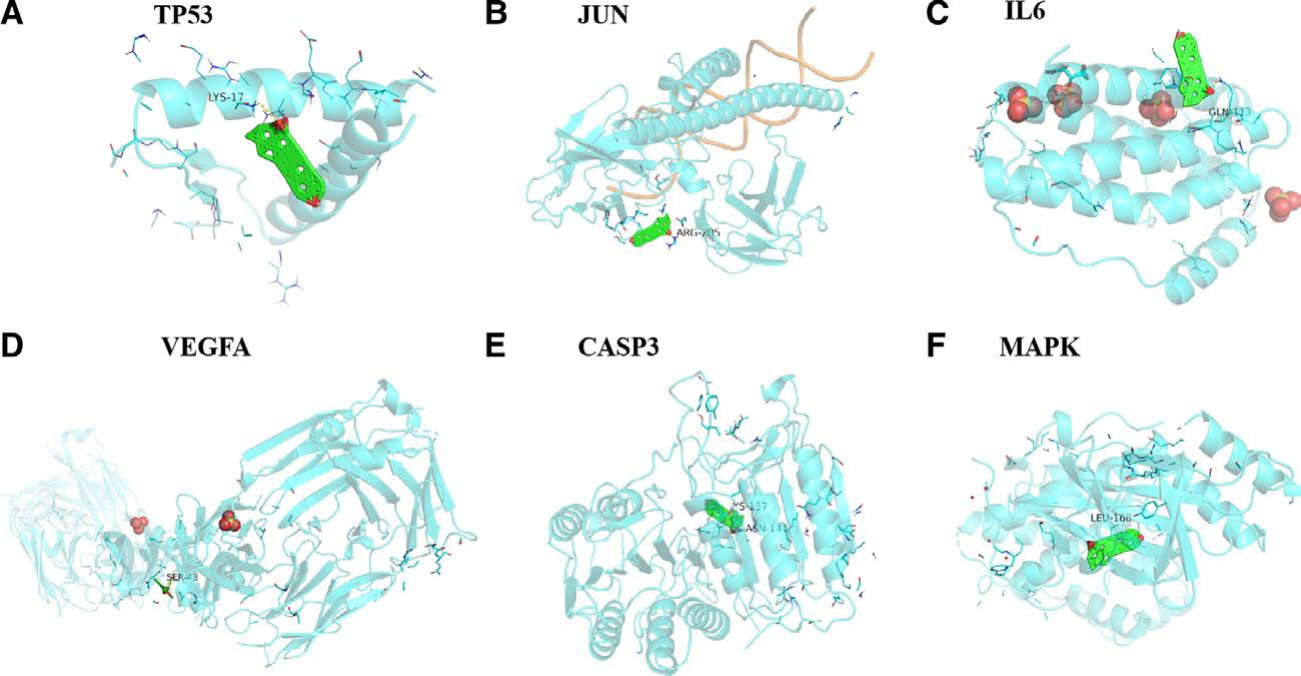
Molecular docking of ursolic acid with 6 core target genes.

The binding energies (docking affinity) of ursolic acid to core target proteins tumor protein p53 (TP53), transcription factor AP-1 (JUN), interleukin- 6 (IL6), vascular endothelial growth factor A (VEGFA), CASP3, and mitogen-activated protein kinase (MAPK8) were −10.5, −9.8, −10.6, −10.6, −10.5, −11.5 kcal/mol (1 kcal = 4.186 kJ), respectively (Table 3). Both of binding energies were lower than −5 kacl/mol, which indicating that ursolic acid has a strong binding activity to the protein by the core target gene. The results of molecular docking between ursolic acid and the protein by the core target gene (the top 6 in binding energy) are shown in Figure 6.

**Table 3:** The results of molecular docking.

Compound	Target	PDB	Energy (kcal/mol)
Ursolic acid	CASP3	1cp3	−10.5
Ursolic acid	IL6	1alu	−10.6
Ursolic acid	MAPK8	1ukh	−11.5
Ursolic acid	JUN	1a02	−9.8
Ursolic acid	TP53	1a1u	−10.5
Ursolic acid	VEGFA	1bj1	−10.6

CASP3 = caspase3, IL6 = interleukin-6, JUN = transcription factor AP-1, MAPK8 = mitogen-activated protein kinase 8, PDB = protein data bank, TP53 = tumor protein p53, VEGFA = vascular endothelial growth factor A.

## 4. Discussion and conclusion

OP is a systemic bone disease characterized by progressive loss of bone mass, fragility, and susceptibility to fractures.^[18]^ According to the results of the first epidemiological survey of OP in China, OP has become an important health problem in the middle-aged and elderly population in my country. The prevalence of OP in people over 50 years old is 19.2%, the prevalence of OP in China has reached 51.6%, which seriously affects the quality of life of the elderly.^[19]^ Therefore, the related research of OP has attracted more and more attention by scholars. The current clinical drug treatment for OP is still dominated by chemical drugs, including estrogen, selective estrogen receptor modulators, bisphosphonates, calcitonin, and bone formation promoters.^[20]^ Previous study showed that the extensive use of chemical drugs causing a certain degree of kidney damage as well as serious side effects such as induction of breast cancer and endometrial cancer, which restricts its clinical application.^[21]^ TCM has significant effects in the treatment of various diseases, and has few side effects, and is especially suitable for the treatment of senile diseases with declining renal metabolic function.^[22]^ Therefore, TCM exhibits broad potential and application prospects in preventing and treating kidney injury and improving OP. Ursolic acid belongs to ursane-type pentacyclic triterpenoids, which exist in natural medicines such as bearberry, privet lucidum, wild rose hip, hedyotis diffusa, plantain, etc. It exhibits various effects biological function which has been used for many years.^[23]^ Compared with a series of adverse reactions such as renal injury caused by chemical drugs in the treatment of OP, ursolic acid has obvious improvement effect on renal injury.^[24]^ It was believed that the kidney stores the essence, and the essence produces the marrow in TCM. The marrow resides in the bone cavity to nourish the bones. When the kidney essence is deficient, the bones wither and the marrow decreases. Therefore, the kidneys play a crucial role in the growth and development of bones in “kidney governing bones” theory.^[25]^ From the above research results and the theory of TCM, it was revealed that ursolic acid has the potential in treatment of OP. In thie present study, a total of 52 ursolic acid-related target genes and 4657 OP-related target genes were excavated. Through venn diagram analysis, 43 common target genes of component-disease were obtained. The PPI network of the above common target genes shows that the target genes are interconnected and interact with each other; the network topology analysis results show that the core target genes of ursolic acid in the treatment of OP mainly include P53, VEGFA, IL6, TNF, CASP3, MMP9, JUN, STAT3, MAPK8, and PTGS2, etc. The occurrence and development of OP is closely related to a variety of inflammatory cytokines. Some studies have pointed out that IL-6 and TNF-α have been confirmed as important inflammatory cytokines involved in bone resorption, which can promote bone resorption and aggravate the disease development of patients.^[26]^ Among them, IL-6 is a multi-effect inflammatory cytokine that may participate in multiple processes such as inflammatory response, immune response, hematopoietic regulation and tumorigenesis.^[27]^ Previous studies have shown that IL-6 may regulate the apoptosis and differentiation of osteoblasts through various signaling pathways, and promote the formation of osteoclasts, thereby promoting bone resorption and ultimately leading to the occurrence of OP.^[28]^ In addition, it was reported that IL-6 may also induce and activate the signal transducer and activator of transcription 3 (STAT3), MAPK and intracellular phosphatidylinositol kinase (PI3K), and these 3 factors are closely related to the occurrence and development of OP.^[29–31]^ It was revealed that TNF-α may not only induce the expression of nuclear factor kappa B (NF-κB) receptor activator ligand (RANKL) in osteoblasts, but also directly act on RANKL to induce the formation of osteoclasts and promote bone resorption.^[32]^ It was found that ursolic acid may protects reduce the levels of cytokines TNF-α and IL-1*β* that against ulcerative colitis via anti-inflammatory and antioxidant effects in mice.^[33]^ The above researches suggests that IL-6 and TNF-α are potential targets of ursolic acid in the treatment of OP.

MAPK8 is a member of the MAPK family and plays an important role in regulating inflammatory and immune responses. On the one hand, MAPK plays an important role in the formation and activation of osteoclasts that affect the development of OP by mediating the NF-κB signaling pathway.^[34]^ On the other hand, activated MAPK may promote the generation of osteoblasts, thus improving the symptoms of OP.^[35]^ Prostaglandins induce macrophage colony-stimulating factor (M-CSF) and RANKL expression, and also promote osteoclast differentiation and inhibit osteocyte function.^[36]^ Prostaglandin endoperoxidase 2 (PTGS2) is the rate-limiting enzyme in the production of prostaglandins and has a certain inhibitory effect on the above-mentioned activities of prostaglandins.^[37]^ Vascular endothelial growth factor (VEGF) both supports angiogenesis and promotes the function of osteoblasts that may partially regulate the functions of osteoblasts and osteoclasts through autocrine, endocrine and paracrine mechanisms, and promote bone formation and reduction of bone resorption.^[38]^ The caspase family is a key factor in regulating the process of apoptosis, among which caspase 3 is one of the most critical apoptotic effectors in this family and is considered to be the executor of apoptosis. When caspase 3 is activated, apoptosis cannot be reversed.^[39]^ In the present study, through the GO functional annotation analysis of shared targets, it was found that GO biological processes were mainly concentrated in the process of apoptosis, regulation of cell proliferation, and drug response. Through the KEGG pathway enrichment analysis of common target genes, it was revealed that they were mainly enriched in tumor pathway, TNF signaling pathway, hepatitis B, PI3K/Akt signaling pathway, human HTLV-I infection and other pathways. Among them, the PI3K/Akt signaling pathway is closely related to bone tissue metabolism.^[40]^ Activation of the PI3K/Akt signaling pathway may stimulate the proliferation and differentiation of osteoblasts while inhibiting their apoptosis. In addition, PI3K can stimulate the formation of osteoclast actin filaments, regulate cell chemotaxis, attachment and proliferation, and inhibit the PI3K expression reduces bone resorption by mature osteoclasts.^[41]^ Previous studies have been confirmed that ursolic acid can inhibit the conduction of PI3K/Akt signaling pathway, resulting in up-regulation of downstream pro-apoptotic protein expression, activation of endoplasmic reticulum stress pathway, and ultimately promoting cell apoptosis.^[42]^ Relevant studies have confirmed that there is a close relationship between chronic hepatitis B virus infection and OP, and its mechanism of action may be related to the chronic inflammation caused by the virus infection.^[43]^ Hepatitis B virus infection can induce the secretion of inflammatory cytokines such as TNF-α, IL-1, and IL-6, which can increase the expression of RANKL and stimulate bone resorption.^[44]^ The combined effect of the above-mentioned inflammatory cytokines is the main cause of OP caused by chronic hepatitis B virus infection, which can lead to decreased bone formation and increased bone resorption, thereby reducing bone mineral density.^[45]^ In addition, HBV infection inhibits hepatic 25-hydroxyvitamin D production and vitamin D uptake, thereby increasing bone loss and reducing bone formation.^[46]^ Hepatic decompensation caused by long-term hepatitis B virus infection affects collagen synthesis in bone matrix by inhibiting fibroblast growth, and may co-suppress osteoblast function by increasing the expression of carcinoembryonic fibronectin.^[47]^

In the present study, molecular docking technology was used to verify the molecular docking between ursolic acid and the encoded proteins of core target genes TP53, JUN, IL6, VEGFA, CASP3, and MAPK8. The results showed that ursolic acid has strong docking activity with the above-mentioned core target gene-encoded protein (the binding energy is less than −5.0kcal/mol), which further indicates that ursolic acid can interact with the core target gene-encoded protein receptor. Stable binding and play its role in the treatment of OP. The results preliminarily verified the reliability of the above-mentioned network pharmacological prediction results, and laid a foundation for the subsequent research on the mechanism of action of ursolic acid in the treatment of OP.

Taken together, the therapeutic effect of ursolic acid on OP may be through the regulation of core target genes such as P53, VEGFA, IL6, TNF, CASP3, MMP9, JUN, STAT3, MAPK8, and PTGS2. It may mainly related to cancer pathways, hepatitis B signaling pathway, TNF signaling pathway and many other key pathways. Its mechanism may involves multiple therapeutic targets and signaling pathways, reflecting the characteristics of TCM for treating diseases with multiple targets and multiple pathways. However, the potential targets and pathways of ursolic acid in the treatment of OP predicted in the present study still need to be further verified by relevant experiments.

## Acknowledgments

Shuqing Zhou and Jingxin Mao conceived and designed the research. Jingxin Mao, Bowen Yang, and Qiuwen Zhu carried out the analysis and wrote the paper in the study. Shuqing Zhou and Jingxin Mao make the equally contribution to the manuscript. All authors declare that there have not any commercial or associative interest that represents competing interests in connection with the work submitted.

## Author contributions

**Conceptualization:** Jingxin Mao, Shuqing Zhou.

**Data curation:** Bowen Yang, Qiuwen Zhu, Jingxin Mao.

**Formal analysis:** Bowen Yang, Xiaodong Wang.

**Funding acquisition:** Jingxin Mao.

**Investigation:** Bowen Yang.

**Methodology:** Bowen Yang.

**Resources:** Qiuwen Zhu, Jingxin Mao, Shuqing Zhou.

**Software:** Qiuwen Zhu, Xiaodong Wang.

**Supervision:** Qiuwen Zhu, Jingxin Mao, Shuqing Zhou.

**Validation:** Qiuwen Zhu, Xiaodong Wang, Jingxin Mao.

**Visualization:** Qiuwen Zhu, Xiaodong Wang, Shuqing Zhou.

**Writing – original draft:** Bowen Yang.

**Writing – review & editing:** Jingxin Mao.
